# Comprehensive analysis of FOXM1 immune infiltrates, m6a, glycolysis and ceRNA network in human hepatocellular carcinoma

**DOI:** 10.3389/fimmu.2023.1138524

**Published:** 2023-05-10

**Authors:** Ziwu Xu, Chaozhu Pei, Haojie Cheng, Kaixin Song, Junting Yang, Yuhang Li, Yue He, Wenxuan Liang, Biyuan Liu, Wen Tan, Xia Li, Xue Pan, Lei Meng

**Affiliations:** ^1^ School of Pharmacy, Hunan University of Chinese Medicine, Changsha, China; ^2^ College of Biology, Hunan University, Changsha, China; ^3^ School of Medical, Hunan University of Chinese Medicine, Changsha, China; ^4^ Department of Pathology, Changsha Hospital of Traditional Chinese Medicine, Changsha Eighth Hospital, Changsha, China; ^5^ Department of General Surgery, People's Hospital of Hunan Province, Changsha, China

**Keywords:** FoxM1, hepatocellular carcinoma, immune infiltration, m6A modification, glycolysis

## Abstract

**Background:**

Forkhead box M1 (FOXM1) is a member of the Forkhead box (Fox) transcription factor family. It regulates cell mitosis, cell proliferation, and genome stability. However, the relationship between the expression of FOXM1 and the levels of m6a modification, immune infiltration, glycolysis, and ketone body metabolism in HCC has yet to be fully elucidated.

**Methods:**

Transcriptome and somatic mutation profiles of HCC were downloaded from the TCGA database. Somatic mutations were analyzed by maftools R package and visualized in oncoplots. GO, KEGG and GSEA function enrichment was performed on FOXM1 co-expression using R. We used Cox regression and machine learning algorithms (CIBERSORT, LASSO, random forest, and SVM-RFE) to study the prognostic value of FOXM1 and immune infiltrating characteristic immune cells in HCC. The relationship between FOXM1 and m6A modification, glycolysis, and ketone body metabolism were analyzed by RNA-seq and CHIP-seq. The competing endogenous RNA (ceRNA) network construction relies on the multiMiR R package, ENCORI, and miRNET platforms.

**Results:**

FOXM1 is highly expressed in HCC and is associated with a poorer prognosis. At the same time, the expression level of FOXM1 is significantly related to the T, N, and stage. Subsequently, based on the machine learning strategies, we found that the infiltration level of T follicular helper cells (Tfh) was a risk factor affecting the prognosis of HCC patients. The high infiltration of Tfh was significantly related to the poor overall survival rate of HCC. Besides, the CHIP-seq demonstrated that FOXM1 regulates m6a modification by binding to the promoter of IGF2BP3 and affects the glycolytic process by initiating the transcription of HK2 and PKM in HCC. A ceRNA network was successfully obtained, including FOXM1 - has-miR-125-5p – DANCR/MIR4435-2HG ceRNA network related to the prognosis of HCC.

**Conclusion:**

Our study implicates that the aberrant infiltration of Tfh associated with FOXM1 is a crucial prognostic factor for HCC patients. FOXM1 regulates genes related to m6a modification and glycolysis at the transcriptional level. Furthermore, the specific ceRNA network can be used as a potential therapeutic target for HCC.

## 1 Introduction

Liver cancer is the third most prevalent malignancy, mainly comprising hepatocellular carcinoma (HCC, also known as LIHC) and intrahepatic cholangiocarcinoma (ICC), of which incidence and mortality rates are increasing worldwide (1). HCC accounts for approximately 75% ~ 90% of all liver cancer cases and is a significant cancer type with a poor prognosis (2). Despite the latest advances in HCC screening and treatment modalities, conventional curative treatments are generally ineffective for HCC because most HCC patients present at an advanced stage to an extent when they are diagnosed (3). Therefore, researching the in-depth investigations of the underlying tumorigenesis and tumor development mechanisms of HCC for screening and prevention is paramount.

The FOXM1 transcription factors are crucial for G1–S and G2–M cell cycle phase progression and mitotic spindle integrity (4). In tumor cells, the expression and the transcriptional activity of FOXM1 are typically upregulated, and overexpression of FOXM1 has been involved in almost all major hallmarks of cancer, manifesting an oncogenic function (5). On the one hand, FOXM1 promotes cancer development by transactivating the expression of its target genes during transcription. On the other hand, FOXM1 may play an oncogenic role that functions within protein interaction networks and protein complexes to activate different oncogenic pathways (6). The strategy of targeting transcription factors has been considered a promising approach in tumor therapy (7). Regarding the multiple biological functions of FOXM1, it has been proven to be a potential therapeutic target for cancer (8), while there are no FDA-approved FOXM1 targeting drugs in oncology treatment.

**Figure 1:**
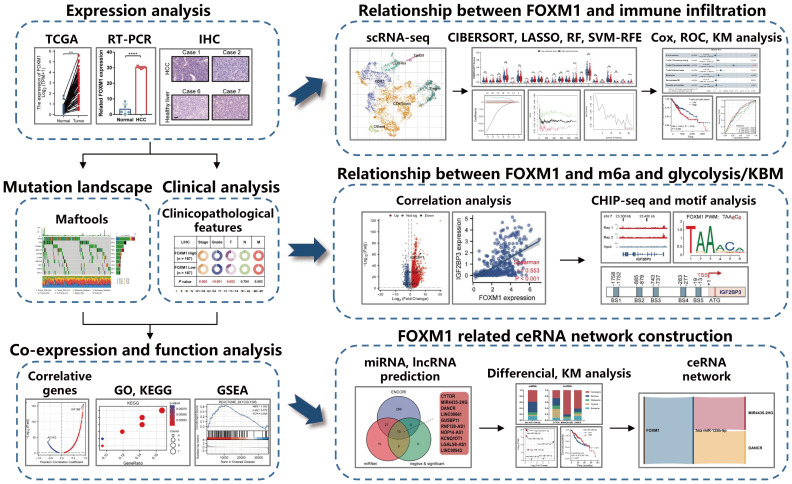
Schematic diagram of the study design.

This study analyzed TCGA data of hepatocellular carcinoma patients for gene RNA-seq expression and clinical information. It used multidimensional analysis to provide an understanding of expression patterns and functional networks to the expression of FOXM1. Use machine learning methods to screen potential diagnostic FOXM1-related infiltrating immune cells in HCC. Evaluating the relationship between differential FOXM1 expression and m6a and glycolysis/KBM-related genes and a comprehensive analysis of genome-wide FOXM1 binding sites in Huh-7 cell lines predicts FOXM1-driven m6a and glycolysis/KBM gene regulation. Finally, FOXM1 was used as the core molecule to predict its associated ceRNA network. To provide a theoretical basis for discovering possible molecular pathways of FOXM1. The schematic diagram of the research design is shown in **Figure 1**.

## 2 Method

### 2.1 Ethics statement

This study proposal has been approved by the Ethics Committee of Changsha Hospitalof Traditional Chinese Medicine (Changsha Elghth Hospltal) and conducted in accordance with the research principles described in the Helsinki Declaration.

### 2.2 Data collection and preprocessing

The GDC download tool (https://portal.gdc.cancer.gov) from the Cancer Genome Atlas (TCGA) database was used to download the transcriptome data and clinical follow-up data for liver cancer (TCGA-liver hepatocellular carcinoma, LIHC). Each gene expression was normalized using the transcripts per kilobase of exon model per Million mapped reads (TPM) metric. Then we kept the expression matrix of 56,494 genes containing 50 normal and 374 tumor samples. The GSE84006 HCC array datasets containing 38 paired samples were downloaded from the gene expression omnibus (GEO) database (https://www.ncbi.nlm.nih.gov/geo/). When multiple probes correspond to the same gene symbol, the maximum value was considered the final value. The somatic variants in Mutation Annotation Format (MAF) were downloaded from TCGA, and Maftools (9) R package was used for the integrative analysis of somatic variants. The CHIP-seq data is a bigwig and bed file of the Huh-7 cell line that was downloaded in the GSE176383 dataset with reference GRch38primary assembly and visualized by IGV software. Every CHIP-seq peak was called at a false discovery rate (FDR) < 0.05.

### 2.3 Differentially expressed genes screening

The original expression data of TCGA were transformed with log2, and differentially expressed genes (DEGs) were identified by the DESeq2 package of R language (9) under the criteria of |log2FC| > log2(2) and *P* < 0.05.

### 2.4 RNA extraction and RT-PCR

The total RNA of patients’ tissues was isolated from cells using RNA easy fast tissue/cell kit (TIANGEN, China, DP451). Use Prime Script RT reagent kit (TIANGEN, China, KR118-02) for reverse transcription, and then use SYBR Prime Script RT PCR kit (TIANGEN, China, FP209-02) for RT-PCR. Use β-Actin as an internal reference and the 2^-△△Ct^ method to calculate the results. FOXM1 primer sequences: forward primer 5’- GCTTGCCAGAGTCCTTTTTGC -3’ and reverse primer 5’- CCACCTGAGTTCTCGTCAATGC -3’. β-Actin primer sequences: forward primer 5’- CATGTACGTTGCTATCCAGGC -3’ and reverse primer 5’- CTCCTTAATGTCACGCACGAT -3’.

### 2.5 Immunohistochemistry

HCC tissues from five patients with HCC and five patients with healthy livers were fixed with 4% paraformaldehyde, dehydrated, paraffin-embedded, and prepared into tissue chips. After dewaxing and hydration, a 10 mM sodium citrate antigen repair solution was used at 95°C for 15 min for antigen repair. Then endogenous peroxidase was blocked by 3% H2O2 for 30 min at room temperature. Nonspecific antigens were blocked with 5% BSA in PBS for 30 min. The mouse monoclonal antibodies of FOXM1 (1:200, Cell Signaling Technology, USA, 20459) were incubated overnight at 4°C. Next, secondary antibody binding was detected with goat anti-mouse IgG-HRP (1:2000, Beyotime, China, A0216). DAB and hematoxylin were then used for staining. Images were photographed with a microscope.

### 2.6 Co-expression analysis

Based on the LinkedOmics platform (10) (http://www.linkedomics.org/admin.php) using the Pearson correlation test method for RNA-Seq, FOXM1 positive and negative co-expression genes were screened under the condition of FDR < 005. The protein-protein interactions PPI network for FOXM1 and its neighbor genes was constructed using the String platform (https://cn.string-db.org/). The R package clusterProfiler (11) was used to process the Genome Ontology (GO) and Kyoto Encyclopedia of Genes and Genomes (KEGG) pathway analysis of FOXM1 co-expression genes.

### 2.7 Gene set enrichment analysis

Based on the clusterProfiler package (11), gene set enrichment analysis (GSEA) (12) was carried out for further underlying mechanism analysis of FOXM1. Patients in the LIHC cohort were stratified into low or high groups based on gene expression, using the median expression as the cut-off value. The high-expression was compared with the low-expression group by GSEA analysis. The c2.cp.v7.2.symbols.gmt [Curated] was selected as annotated gene set.

### 2.8 Machine learning and immune infiltration analysis

To uncover the potential role of FOXM1 on the single-cell level, the Tumor Immune Single-cell Hub (TISCH) database (13) (http://tisch.comp-genomics.org/home/) was employed to analyze the correlation between FOXM1 expression and immune cells. Furthermore, We utilized the Least absolute shrinkage and selection operator (LASSO), random forest (RF), and support vector machine-recursive feature elimination (SVM-RFE) to figure out the key immune cells from 22 immune cells by the CIBERSORT (14) algorithm. First, we performed the CIBERSORT algorithms to quantify immune cells’ activity or enrichment levels in LIHC tumor tissues. Subsequently, the “glmnet,” “randomForest,” and “e1071” R package was performed with the CIBERSORT scores of FOXM1 high and low group and follow-up data of each patient to carry out the LASSO, SVM-RFE, and RF analysis of immune cells, respectively. The overlapping immune cells of three algorithms were further screened with multivariate Cox regression analysis, and the key immune cells were evaluated by ROC curve.

### 2.9 CeRNA network analysis

The multiMiR R package was used for exploring microRNA (miRNA) that has been experientially validated to interact with FOXM1. The long non-coding RNA (lncRNA) interaction with screened miRNA was predicted by ENCORI (15) platform (https://rna.sysu.edu.cn/encori/index.php) and miRNet (16) platform (https://www.mirnet.ca/). Besides, the subcellular localization of ceRNA was analyzed by the lncLocator (17) platform. According to the ceRNA hypothesis, mRNA and lncRNA negatively correlate to miRNA, RNA expression analysis, and overall survival analysis in the LIHC cohort to construct the ceRNA network.

### 2.10 Statistical analysis

All statistical analyses in this study were conducted using GraphPad Prism 7.0 and R. The correlation between diagnostic gene expression levels and clinical factors was determined using unpaired Student’s t-tests for continuous variables and Fisher’s exact tests for categorical variables. To analyze the data normalization, a non-parametric statistical analysis was performed. Data with non-parametric characteristics were analyzed with the Kruskal-Wallis or Wilcoxon two-sample test. A two-sided P < 0.05 was considered to indicate statistical significance for all analyses.

## 3 Result

### 3.1 FOXM1 expression and mutation analysis

**Figure 2:**
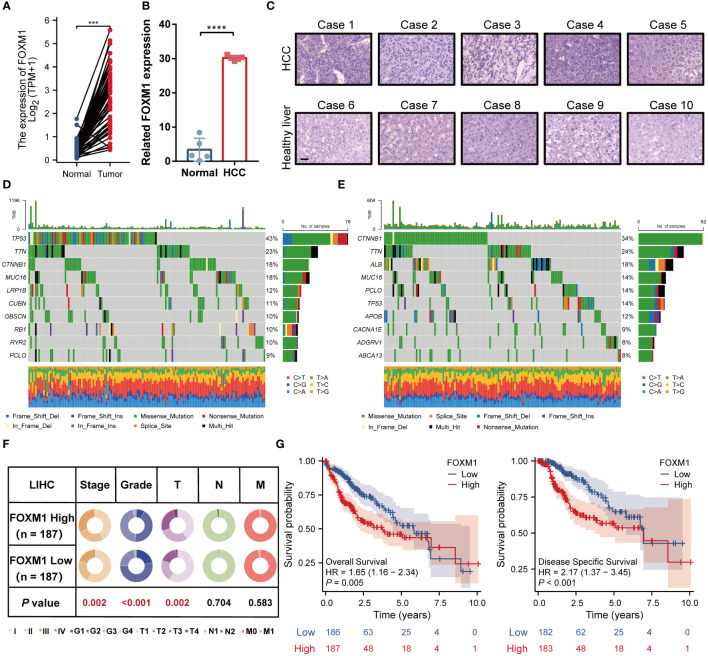
FOXM1 is highly expressed in HCC and is associated with high-frequency mutation and poor prognosis. **(A)** FOXM1 expression level in TCGA databases. **(B)** Difference of expression of FOXM1 in clinical samples. **(C)** The protein level of FOXM1 in healthy liver and primary HCC samples was detected by immunohistochemistry, bar 10 μm. The landscape profile of the top 10 mutated genes in the FOXM1 high expression group **(D)** and FOXM1 low expression group **(E)** from the TCGA database. **(F)** The proportion difference of clinical indices (including tumor, node, metastasis stages, and pathological grade) in the FOXM1 high and low expression groups from the TCGA LIHC dataset. **(G)** Prognostic relationship (OS and DFS) between FOXM1 and patients with HCC. (***p < 0.001, ****p < 0.0001).

The progression of HCC is usually accompanied by abnormal gene expression and poor prognosis. Expression of FOXM1 in matched HCC tissues was found to be higher than that in adjacent samples **(** **Figure 2A** **)**. Data from the RT-PCR revealed a consistent trend (**Figure 2B**). IHC analysis demonstrated that FOXM1 was mainly overexpressed in the nucleus, consistent with its role as a transcription factor **(** **Figure 2C** **)**. We used the maftools package to screen the 10 genes with the highest mutation frequencies in the FOXM1 high and low expression groups, respectively **(** **Figures 2D, E** **)**. The results showed a high frequency of mutations in TP53, TTN, CTNNB1, MUC16, and PCLO in the high FOXM1 expression group. These mutated genes are known biomarkers of HCC and are of great value for evaluating the malignant tumor progression or therapeutic response (18). The critical genes in HCC progression were usually correlated with cancer stages and patient prognosis. Patients with HCC showed more advanced stage, grade, and T stage in the FOXM1 high-expression group **(** **Figure 2F** **)** and shorter overall and disease-free survival **(** **Figure 2G** **)** outcomes.

### 3.2 Enrichment analysis of FOXM1 co-expressed genes

**Figure 3:**
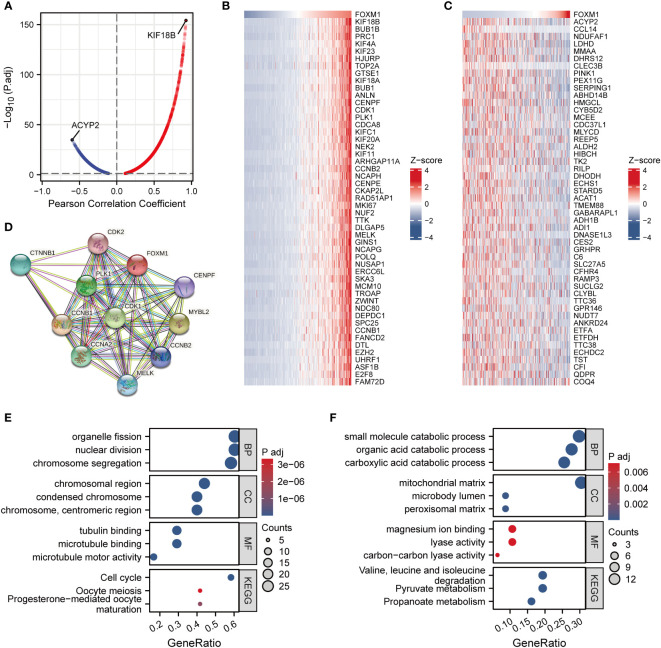
Co-expression network analysis and enrichment analysis of FOXM1 in LIHC. **(A)** Volcano map of correlative genes of FOXM1. **(B)** Heat map of Top50 positive co-expression genes with FOXM1. **(C)** Heat map of top50 negative co-expression genes with FOXM1. **(D)** PPI network analysis of FOXM1. Gene ontology and KEGG analysis for genes belonging to the co-expression with strongest positive **(E)** and negative correlation **(F)** with FOXM1.

To better understand the biological function of FOXM1, we obtained the correlation between each gene and FOXM1 using the LinkedOmics database and analyzed the enrichment of the top 100 genes. There were 8027 genes positively correlated with FOXM1, and 3610 genes negatively correlated with FXOM1 under FDR < 0.05 (**Figure 3A** **)**. The heat map shows the top 50 positively **(** **Figure 3B** **)** and negatively **(** **Figure 3C** **)** associated genes with FOXM1, respectively. Kinesin family member 18B (KIF18B) expression was positively correlated with the expression of FOXM1, suggesting FOXM1 may have similar regulatory functions to KIF18B. Acylphosphatase 2 (ACYP2) had the highest negative correlation coefficient, probably because it plays opposite roles to FOXM1 in different functional pathways. We further performed the STRING database to study the protein-protein interaction (PPI) network of FOXM1 (**Figure 3D**). For the more biological function of FOXM1, Gene Ontology (GO) and Kyoto Encyclopedia of Genes and Genomes (KEGG) enrichment analysis was performed. The result indicated that those genes positively correlated with FOXM were positively associated with the cell cycle and cell division (**Figure 3E**). In contrast, genes negatively associated with FOXM1 in HCC samples were more related to various metabolic pathways (**Figure 3F**).

### 3.3 Gene set enrichment analysis of FOXM1

**Figure 4:**
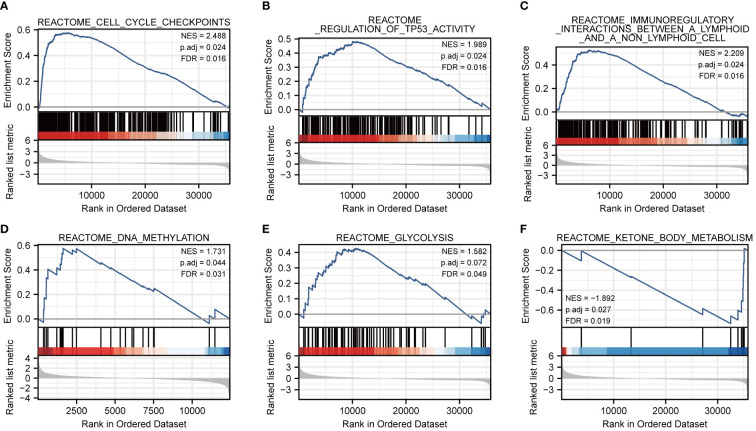
Co-expression network analysis and enrichment analysis of FOXM1 in LIHC. **(A)** Cell cycle checkpoints. **(B)** Regulation of TP53 activity. **(C)** Immunoregulatory interactions between a lymphoid and a non-lymphoid cell. **(D)** DNA methylation. **(E)** Glycolysis. **(F)** Ketone body metabolism.

To further characterize the potential function of FOXM1, GSEA was performed. We separated the LIHC cohort samples into a high-expressed group and a low-expressed group according to the expression level of FOXM1 to identify the gene sets associated with FOXM1. The GSEA results showed that cell cycle checkpoints (FDR = 0.016), regulation of TP53 activity (FDR = 0.016), and immunoregulatory interactions between a lymphoid and a non-lymphoid cell (FDR = 0.016) were upregulated in FOXM1 high-expression cluster. On the contrary, the low-expressed FOXM1 group was enriched for genes implicated in DNA methylation (FDR = 0.031), glycolysis (FDR = 0.049), and ketone body metabolism (FDR = 0.019) (**Figure 4**). Sustaining proliferative signaling, deregulating cellular metabolism, and avoiding immune destruction are the hallmarks of cancer (19). According to GSEA results, we further evaluated the association of FOXM1 in immune infiltration and glycolysis and KBM.

### 3.4 Correlations of FOXM1 expression with immune infiltration

**Figure 5:**
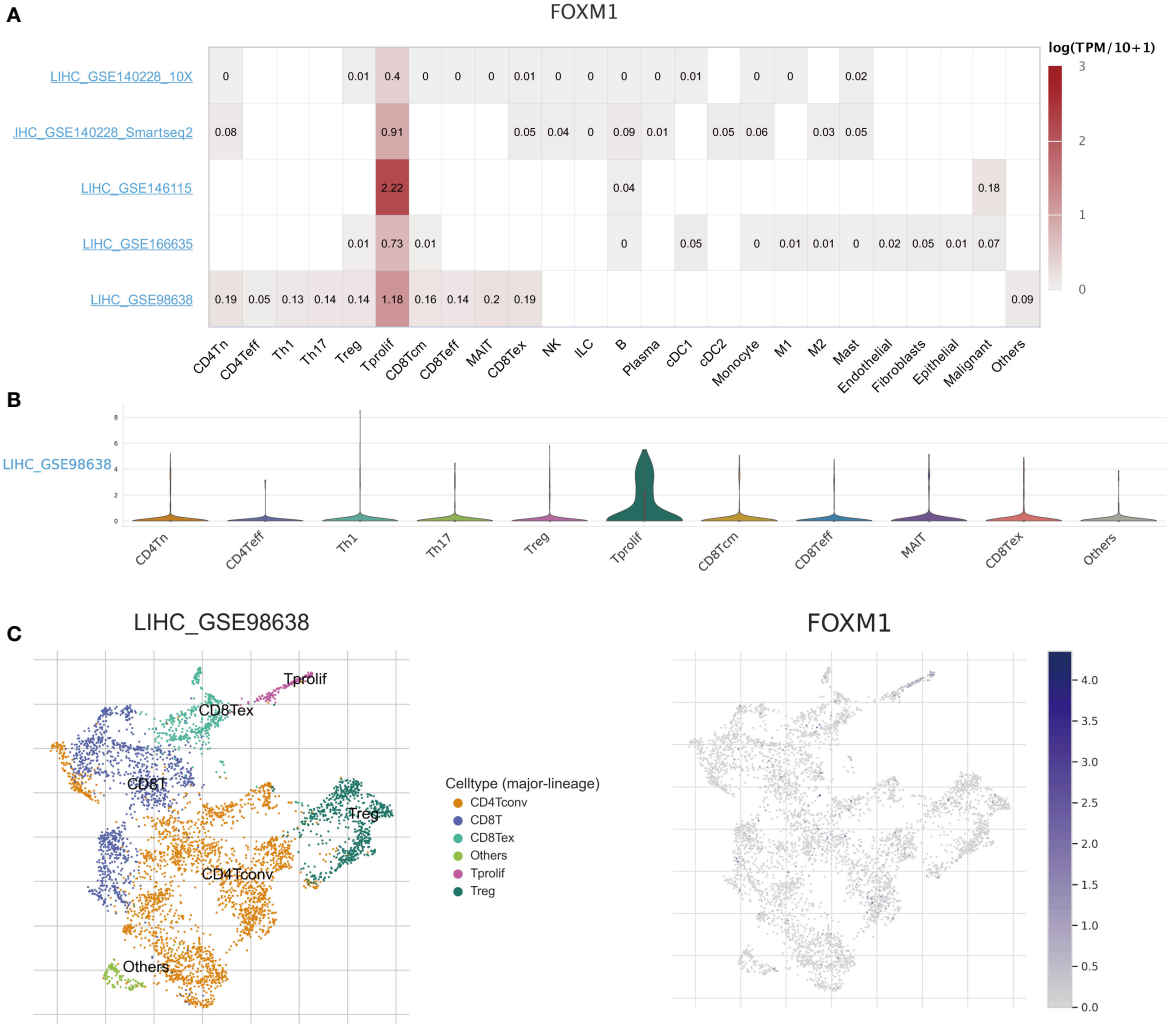
The scRNA-seq analysis of the expression of FOXM1 in different immune cells of HCC. **(A)** Heatmap of the correlation of FOXM1 with immune cell infiltration levels in the independent scRNA-seq database. **(B)** Violin plot of FOXM1 and immune cell infiltration. **(C)** Single-cell atlas of all cells in GSE98638 dataset and the expression and distribution of FOXM1.

Based on the scRNA-seq TISCH database, we obtained five independent HCC datasets for single-cell analysis to explore the correlation between immune cell distribution and FOXM1 expression levels at the single-cell level **(** **Figure 5A** **)**. In the LIHC_GSE98638 dataset, higher levels of FOXM1 expression were found in proliferating T cells (T prolif) **(** **Figures 5A, B** **)**. The distribution and expression of FOXM1 in different immune cells were obtained from the clustered plots of scRNA-seq **(** **Figure 5C** **)**. Those results indicated that FOXM1 expression levels were significantly correlated with immune cell types and their proportions in HCC.

### 3.5 Identification of key immune cells associated with FOXM1

**Figure 6:**
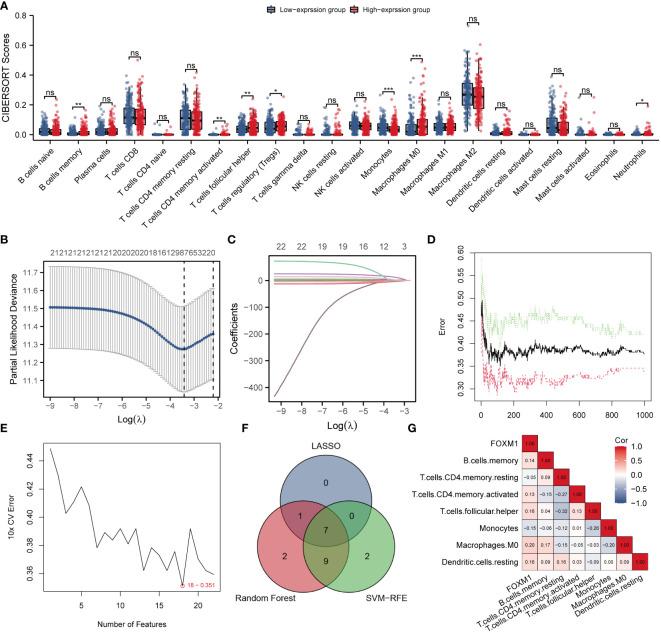
Identification of the key infiltrated immune cells by machine learning. **(A)** Differences in immune infiltration between FOXM1 high and low expression groups. **(B)** Penalty plot of 22 immune cells in the LASSO model, error bars represent standard error. **(C)** Distribution of LASSO coefficients for 22 immune cells. **(D)** The error variation of the RF algorithm, red and green represent the error rate of high and low FOXM1 expression groups, and black represents the overflow error rate. **(E)** Identification of 22 immune cells through the SVM-RFE algorithm. **(F)** 7 immune cell types were identified by LASSO and RF algorithms. **(G)** Correlation graph between the infiltration level of 7 immune cells and the expression level of the FOXM1 gene. (*p < 0.05, **p < 0.01, ***p < 0.001, ns not significant).

Immune infiltration profoundly affects tumor progression, and the composition of tumor-infiltrating immune cells has been identified as a critical factor influencing tumor therapy success (20). The CIBERSORT deconvolution algorithm obtains the percentage of infiltration of 22 immune cells in each HCC sample. Grouping comparison of FOXM1 expression showed that there were seven different types of infiltrating immune cells, namely B cells memory (p < 0.01), T cell CD4 memory activated (p < 0.01), Tfh cells (p < 0.01), T cell regulation (Tregs) (p < 0.05), monocytes (p < 0.001), macrophage M0 (p < 0.001), and neutrophils (p<0.05) (**Figure 6A**). The following study performed LASSO, RF, and SVM-RFE analysis on 22 immune cell infiltration. The penalty parameter was tuned by 10-fold cross-validation in LASSO logistic regression, which selected eight immune cells as the feature **(** **Figures 6B, C** **)**. The RF diagnosis model was developed with ntree = 500 and mtry = 6 and obtained 19 key immune cells **(** **Figure 6D** **)**. Besides, the SVM-RFE algorithm was applied to identify the best feature of the immune cell combination **(** **Figure 6E** **).** Overall, marker genes acquired based on the above three algorithms were intersected to obtain seven key immune cells (B cells memory, T cells CD4 memory resting, T cells CD4 memory activated, Tfh cells, monocytes, macrophages M0, and dendritic cells resting) were selected for subsequent analysis **(** **Figure 6F** **)**. The correlation analysis showed FOXM1 expression is negatively correlated with T cells CD4 memory resting (Cor = -0.05), monocytes (Cor = -0.16), and significantly positively correlated with B cells memory (Cor = 0.14), T cells CD4 memory activated (Cor = 0.13), Tfh cells (Cor = 0.18), macrophages M0 (Cor = 0.20), and dendritic cells resting (Cor = 0.18) **(** **Figure 6G** **)**.

### 3.6 The clinical manifestations of key immune cells

**Figure 7:**
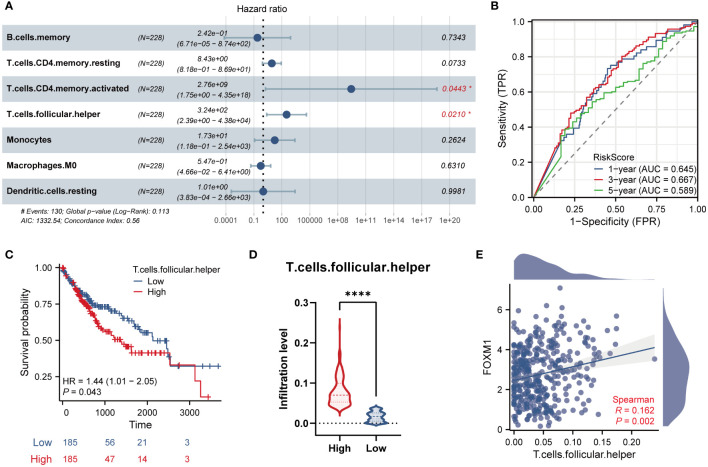
Validation and survival analysis of the key infiltrated immune cells. **(A)** Multivariate Cox Forest plots related to the prognosis of 7 key immune cells. **(B)** The AUC values of the time ROC curves. **(C)** The Kaplan-Meier curve of Tfh cells infiltration level and overall survival. **(D)** The violin plot of Tfh cells infiltration in FOXM1 high and low expression groups. **(E)** The scatter plot of the correlation between the infiltration level of Tfh cells and the FOXM1 expression. (*p < 0.05, ****p < 0.0001).

We further included seven immune characteristics in the multivariate Cox regression. The results showed that T cells CD4 memory resting and Tfh cells were the key immune landscapes associated with FOXM1 **(** **Figure 7A** **)**. We then performed the time ROC analysis to clarify the specificity and sensitivity of the key immune cells. The areas under the curve (AUCs) of 1, 3, and 5-year OS were 0.645, 667, and 0.589, indicating that the prediction model was credible **(** **Figure 7B** **)**. We further investigated the prognostic ability of immune cells associated with FOXM1. We performed Kaplan-Meier analysis on FOXM1 associated immune cells and found that the low infiltration of Tfh cells was significantly associated with the poor prognosis of HCC patients **(** **Figure 7C** **)**. Tfh cell infiltration in the high-expression group was considerably higher than in the FOXM1 low-expression group **(** **Figure 7D** **)**. Furthermore, the Tfh cells infiltration significantly correlates with the FOXM1 expression **(** **Figure 7E** **)**. Taken together, the abnormal infiltration of Tfh cells associated with FOXM1 is a critical factor in predicting the prognosis of HCC patients.

### 3.7 Correlations of FOXM1 expression with m6a-related genes

**Figure 8:**
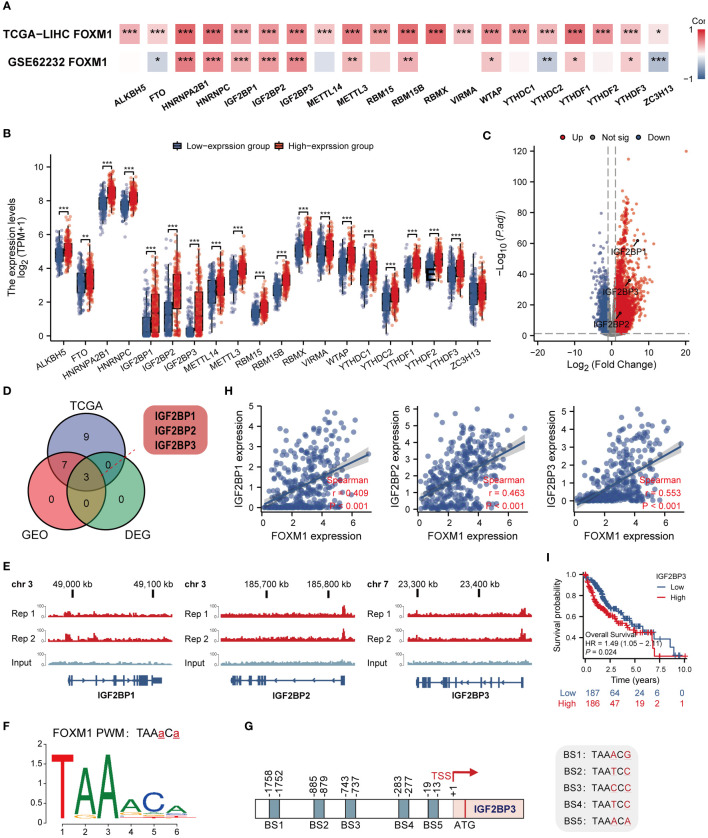
FOXM1 is highly correlated with m6a-related genes and has the ability to potentially transcribe IGF2BP1, IGF2BP2 and IGF2BP3. **(A)** Heat map of the correlation between FOXM1 and m6a-related genes expression. **(B)** Differences in m6a-related gene between FOXM1 high and low expression groups. **(C)** Volcano plots of mRNA that were differentially expressed between HCC and normal tissues. **(D)**Venn diagram of m6A-related genes positively associated with FOXM1 and DEGs in HCC. **(E)** Genome browser screenshots of the FOXM1 binding sites. The test group has two replicates, and the peaks shown are statistically significant. **(F)** Map of FOXM1 binding site sequence. **(G)** Schematic illustration of the potential FOXM1 binding sites (BS) in the IGF2BP3 promoter. **(H)** The scatter plot shows the correlation between the overlapping genes and FOXM1. **(I)** Kaplan-Meier overall survival curves of IGF2BP3. (*p < 0.05, **p < 0.01, ***p < 0.001).

Modifying m6a is a reversible chemical modification on mRNA, which regulates gene expression, controls many cellular and biological processes, and is implicated in human carcinogenesis (21). We first analyzed the TCGA LIHC cohort and GSE62232 data sets to evaluate the correlations between the expression of FOXM1 and m6a-related genes in LIHC **(** **Figure 8A** **)**. The result shows that FOXM1 expression was significantly positively correlated with most m6a-related genes, including HNRNPA2B1, HNRNPC, IGF2BP1, IGF2BP2, IGF2BP3, METTL3, RBM15B, WTAP, YTHDF1 and YTHDF3 (p < 0.001). To determine whether m6a modifications differ from changes in FOXM1 expression, we assessed the relationships between the FOXM1 high and low groups. The result showed that compared with the low expression group, all the m6a-related genes’ expression increased in the high expression group except ZC3H13 **(** **Figure 8B** **)**. By differential analysis, we obtained the m6a-related genes IGF2BP1, IGF2BP2, and IGF2BP3 that are upregulated in HCC **(** **Figure 8C** **)**. After that, among the identified DEGs above, three genes were positively correlated with FOXM1, shared by the TCGA and GEO datasets **(** **Figure 8D** **)**. To corroborate the function of FOXM1 in regulating m6a, the following CHIP-seq analysis was performed in Huh-7. In the analysis of overlapping m6a-related genes, we found that FOXM1 was enriched in the promoter region of IGF2BP1, IGF2BP3, and IGF2BP3 genes **(** **Figure 8E** **)**. Based on the motif sequence of FOXM1 **(** **Figure 8F** **)** predicted by the FootprintDB database (22) (https://footprintdb.eead.csic.es/index.php), five FOXM1 binding sites (BS1, - 1752 to - 1758; BS2, - 879 to -885; BS3, - 737 to – 743; BS4, -277 to - 283 and BS5, - 13 to - 19) were analyzed to exist in the IGF2BP3 promoter **(** **Figure 8G** **)**. Besides, there was a high correlation between FOXM1 and IGF2BP1, IGF2BP3, and IGF2BP3 **(** **Figure 8H** **)**. Kaplan-Meier survival curve demonstrates the prognostic value of IGF2BP3 **(** **Figure 8I** **)**. These results laterally reflect the biological activity of FOXM1 in regulating the transcriptional level of m6a-related genes.

### 3.8 Correlations of FOXM1 expression with glycolytic/KBM-related genes

**Figure 9:**
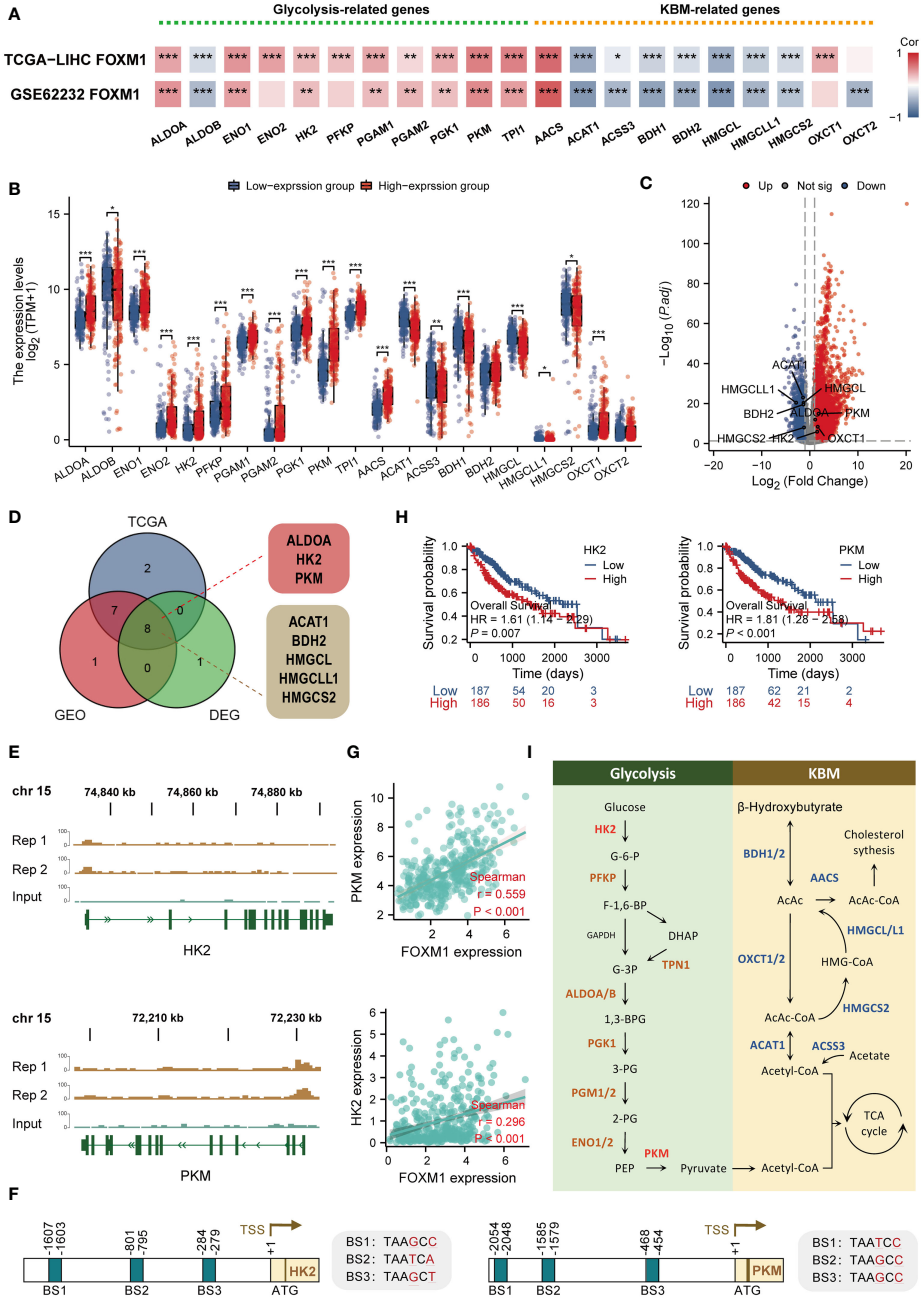
FOXM1 is highly correlated with glycolysis/KBM-related genes and has the ability to potentially transcribe HK2 and PKM. **(A)** Heat map of the correlation between FOXM1 and glycolysis/KBM-related gene expression. **(B)** Differences in glycolysis/KBM-related genes between FOXM1 high and low expression groups. **(C)** Volcano plots of mRNA that were differentially expressed between HCC and normal tissues. **(D)**Venn diagram of glycolysis/KBM-related genes positively associated with FOXM1 and DEGs in HCC. **(E)** Genome browser screenshots of the FOXM1 binding sites. The test group has two replicates, and the peaks shown are statistically significant. **(F)** Schematic model illustrating the glycolysis and KBM pathway. **(G)** The scatter plot shows the correlation between the overlapping genes and FOXM1. **(H)** Kaplan-Meier overall survival curves of HK2 and PKM. **(I)** Diagram showing the position of the recognized promoter sites in FOXM1. The selected signature genes are marked in the pathway, with glycolytic enzymes in brown, ketone bodies metabolic genes in blue, and the FOXM1-bindin genes highlighted in red. (*p < 0.05, **p < 0.01, ***p < 0.001).

Based on pathway enrichment analysis, FOXM1 is involved in a range of metabolic pathways in LIHC. The current study analyzed the correlation of glycolytic and ketone body metabolism (KBM) related genes with FOXM1 expression. Glycolysis and ketone bodies metabolic related genes were manually retrieved from the Molecular Signatures Database v 7.1 (MSigDB) (23). By analyzing the TCGA LIHC cohort and GSE62232 data sets, the correlations between the expression of FOXM1 and glycolytic-related genes were significantly positively correlated, including ALDOA, ENO1, HK2, PGAM1, PKM, TPI1, and as for KBM-related genes (**Figure 9A**). The KBM related genes was ACAT1, ACSS3, BDH1, BDH2, HMGCL, HMGCLL1, HMGCS2 significant negative correlated with FOXM1 (**Figure 9A**). Moreover, we further analyzed the differential expression of glycolytic/KBM-related genes between the high and low expression of FOXM1 (**Figure 9B**). The result showed that compared with the low expression group, the expression of glycolysis-related genes, including ALDOA, ENO1, ENO2, HK2, PFKP, PGAM1, PGAM2, PGK1, PKM, TPI1, and KBM-related genes were increased in the high-expression group. In contrast, there was a decrease in the high-expression group of KBM related genes, including ACAT1 ACSS3 BDH1 HMGCL, HMGCLL1, and HMGCS2. Volcano plots revealed that glycolytic-related genes were upregulated, and more KBM-related genes were downregulated in HCC (**Figure 9C**). We next matched the DEGs to the positive correlation genes in the correlation result of **Figure 9A**. In summary, seven genes were overlapping (**Figure 9D**). Subsequent CHIP-seq analysis revealed significant peaks of FOXM1 in the HK2, and PKM gene promoter regions, suggesting that FOXM1 is involved in the transcription of these genes in Huh-7 cells (**Figure 9E**). Moreover, the motifs of three FOXM1 binding sites were detected in the promoters of HK2 and PKM genes, respectively (**Figure 9F**). The scatter plot shows the correlation between glycolytic/KBM-related genes (**Figure 9G**). Together with Kaplan-Meier curve analysis, HK2 and PKM were significantly relevant with poor prognosis of HCC **(** **Figure 9H** **).** These 21 genes were involved in transforming this substance on the metabolic pathways, among which HK2 and PKM participated in the reaction process of glucose to Glucose-6-phosphate (G-6-P) and phosphoenolpyruvate (PEP) to pyruvate as metabolic enzymes, respectively (**Figure 9I**).

### 3.9 Construction of FOXM1-related ceRNA network

**Figure 10:**
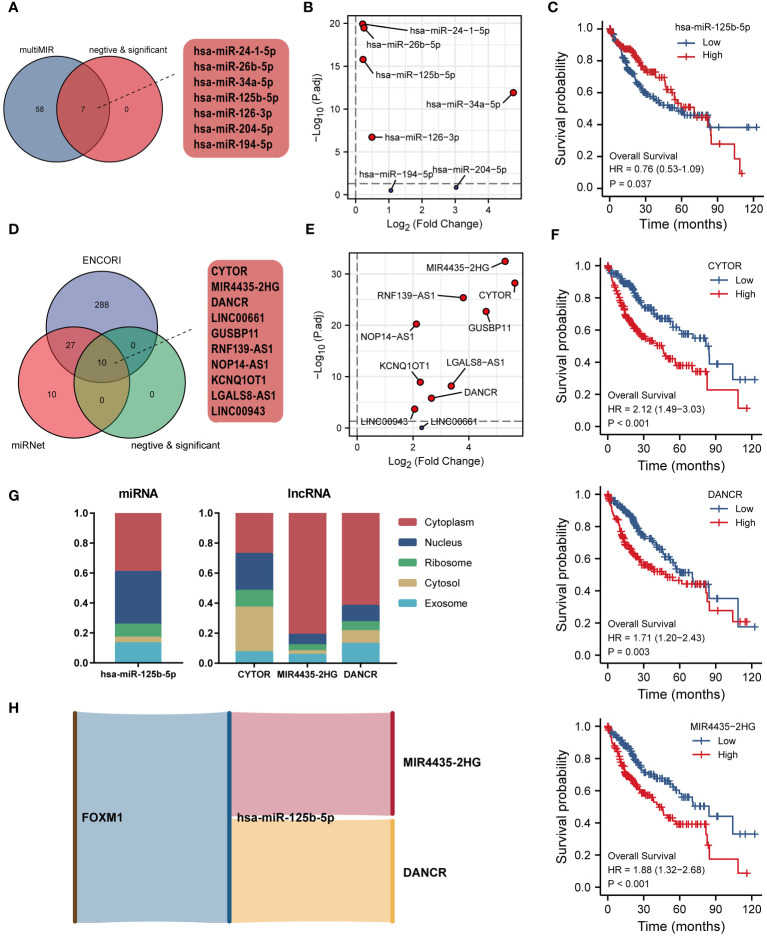
Construction of ceRNA network of FOXM1 in LIHC. **(A)** Venn diagram of miRNA interacting with FOXM1. **(B)** Differential expression of interacted miRNA in TCGA LIHC cohort. **(C)** Kaplan-Meier curve of hsa-miR-125b-5p. **(D)** Venn diagram of lncRNA interacting with hsa-miR-125b-5p. **(E)** Differential expression of interacted lncRNA in TCGA LIHC cohort. **(F)** Kaplan-Meier curve of CYTOR, DANCR, MIR4435-2HG. **(G)** The subcellular localization of screened miRNA and lncRNA. **(H)** Sankey diagram of the ceRNA (mRNA-miRNA-lncRNA) network.

The hypothesis of ceRNA that is involved in tumorigenesis has been validated by various experiments (24). In this study, we carried out an analysis of the FOXM1-related ceRNA network in LIHC. Based on the multiMIR R package, 65 experimentally validated miRNA interacting with FOXM1 had been retrieved. Since miRNA expression and ceRNA (mRNA, lncRNA, etc.) expression take on a negative trend according to the ceRNA hypothesis, there were 5 targeting miRNA that had been screened in the matched miRNA (**Figure 10A**). The significantly expressed miRNA had been identified through the transcript-level differential analysis expression profiles of the selected miRNA integrated from the TCGA project in paracancerous and hepatic tumor tissue (**Figure 10B**). Kaplan-Meier analysis revealed that only hsa-miR-125b-5p low-expressed played a significant role in LIHC patients’ poor prognosis (**Figure 10C**). Further, the lncRNA interaction with miRNA was predicted, and the Venn diagram demonstrates the interacting lncRNA by retrieving the differentially expressed miRNA from the ENCORI and miRNet database (**Figure 10D**). Followed difference analysis of the lncRNA that significantly negatively correlated with miRNA in the database intersection was also done (**Figure 10E**). We consistently utilized Kaplan-Meier analysis on the screened lncRNA in the ceRNA network, finding that CYTOR, DANCR, and MIR4435-2HG were related to prognosis (**Figure 10F**). Besides, the subcellular localization of ceRNA components can be a possible influential factor in ceRNA activity and contributes to human diseases, including cancer (24). Since the ceRNA network mainly exists in the cytoplasm, we further analyzed the lncRNA cellular distribution by performing the lncLocator platform (**Figure 10G**). Predicting that MIR4435-2HG and DANCR were mainly distributed in the cytoplasm, but CYTOR was located primarily on cytosol. These data indicated that the FOXM1-related lncRNA-miRNA-mRNA triple regulatory networks constructed from the RNAs, which MIR4435-2HG and DANCR act as ceRNAs to improve the expression of FOXM1 through sponging hsa-miR-125b-5p (**Figure 10H**).

## 4 Discussion

The FOX gene family is an evolutionarily conserved gene family that encodes approximately 50 transcription factors in the human genome (25). These critical transcription factors broadly regulate gene transcription and involve various biological processes (26). FOXM1 is the only member of the FOXM subfamily known for its functionality in promoting the G1-S and the G2-M cell cycle transition (27). It is mainly detected along with the growth of cells (5). Additionally, FOXM1 is crucial in tumor development and is associated with poor prognosis (28). Current studies have identified FOXM1 as a tumor-specific biomarker with powerful predictive prognostic capacity in HCC (29, 30). In this study, FOXM1 is highly expressed in HCC, related to advanced TNM staging and poor prognosis, consistent with the study (31). We analyzed the relationship between the FOXM1 expression and the prognosis, survival rate, tumor stage, and lymph node metastasis of HCC patients through TCGA data. HCC patients with high FOXM1 expression have a low survival rate significantly related to lymph node metastases and HCC clinical stage.

FOXM1 was first identified as a protein that regulates cell cycle and proliferation (32). While with the developed understanding of cancer mechanisms, novel hallmarks have been further expanded (33). In the analysis of FOXM1 co-expression in RNA-seq data from HCC patients, KIF18B had a positive correlation with FOXM1. As reported, KIF18B is a molecular motor protein that destabilizes astral microtubules during mitosis (34), which promotes tumor development in various cancers and is associated with poor prognosis (35). Moreover, KIF18B and FOXM1 similarly mediate DNA double-strand break repair (34) and participate in the cell cycle and DNA replication (36). KIF18B is also closely associated with infiltrating immune cells (37). These results imply that FOXM1 may have similar biological functions to KIF18B. Research reports ACYP2, which we found is negatively associated with FOXM1, whose polymorphisms are related to changes in plasma telomerase levels (21). The SNPs of ACYP2 can serve as risk and protective factors in HCC, respectively (38). However, the discussion on the relationship between the ACYP2 gene and cancer is still relatively scarce. In turn, enrichment analysis was performed of the gene set. The positively correlated group had several significantly enriched categories, including cell cycle and cell division. In contrast, the negatively correlated group enriched genes related to the catabolic process and metabolism. Besides, the GSEA of FOXM1 indicated that the pathways of cell cycle checkpoints, regulation of TP53 activity, immunoregulatory interactions between a lymphoid and a non-lymphoid cell, DNA methylation, glycolysis were up-regulated, and downregulation of ketone body metabolism. These results suggest that FOXM1 may exert metabolic and immune biological functions in the process of cancer. In this article, we focus on the relationship of FOXM1 in immune infiltration, m6a modification, and glycolysis/KBM, as previous studies have associated these pathways with HCC progression and metastasis.

The immune cells in the tumor microenvironment have a complex biological relationship with tumor cells and may lead to tumor development or suppression, resulting in differential immunotherapeutic responses (39). In scRNA-seq analysis, we evaluated the association between infiltrating immune cells and FOXM1 in HCC tissues and found that FOXM1 was mainly distributed in T prolif cells. The relationship between FOXM1 and the promotion of B cell proliferation (40) and induction of M2 macrophage polarization (41) in non-cancerous conditions has been reported. FOXM1 has also been found to recruit macrophage migration in FOXM1 in lung cancer (42). If only the biological functions of FOXM1 in the cell cycle and cell proliferation are considered, it is not surprising that FOXM1 is predominantly expressed in T-proliferating cells. However, FOXM1 does not solely promote immune cell infiltration in tumors; it also can suppress the maturation of BMDCs *via* direct activation of Wnt5a and weakened promotion of T-cell proliferation (43). Therefore, it still suggests the relevance of FOXM1 to immune infiltration and the bias of FOXM1 expression in different types of infiltrating immune cells. Besides, based on the CIBERSORT algorithm, FOXM1 expression was positively correlated with T cell CD4 memory activated, Tfh cells, and Tregs in T cell subsets infiltration. Given the complex relationships between immune cells in the tumor microenvironment, we predicted the immune cells with prognostic value. Further machine learning (LASSO, RF, and SVM-RFE algorithms) analysis found that seven immune infiltrating cells were significantly associated with patients with hepatocellular carcinoma in the FOXM1 expression subgroup. Cox regression and Kaplan-Meier analysis showed that high invasion of Tfh cells was associated with poor prognosis in HCC patients.

Tfh cells are a specific subpopulation of CD4^+^ T cells that help B lymphocytes produce an adequate antibody response to various pathogens (44). Nonetheless, Increasing evidence for the increase of Tfh cells was associated with poor prognosis (45, 46), and the rate of Tfh cell infiltration is higher in late-stage patients than in early-stage patients. Tfh cells also have known roles in the origin of T cell malignancies and assist malignant B cells (47). Besides, Tfh cells exacerbate immune-related adverse events, such as immune checkpoint blockade (ICB) and autoimmunity during cancer immunotherapy (48). Research on FOXM1 and immune regulation has been reported. FOXM1 selectively upregulates PD-L1 expression by directly binding to the PD-L1 promoter in the nucleus (49). High glucose microenvironment can activate CD27 transcription and expression in CD8^+^ T cells *via* the mTOR-FOXM1 pathway, thereby significantly enhancing the immunocidal effect of CD8^+^ T cells (50). This study also found a correlation between FOXM1 and immune cell infiltration. Moreover, the abnormal infiltration of the Tfh cells associated with FOXM1 may be a key predictor of HCC based on various machine-learning algorithms.

Regarding m6a modification, the most abundant mRNA modification plays different roles in various biological processes by affecting gene expression post-transcriptionally in eukaryotes (51). The m6a modification is a highly dynamic and reversible process involving enzymes responsible for the installation of modifications called “Writers,” the removal of methylation called “Erasers,” and the recognition of modifications called “Readers” (52). However, m6a is often dysregulated in various types of cancer, leading to tumorigenesis, progression, and metastasis (53). Therefore, understanding the correlation between m6a modification of FOXM1 is rewarding to understanding the regulation mechanism of FOXM1 in LIHC. This study found that most m6a-related genes were positively correlated with FOXM1 expression, and IGF2BP family proteins (IGF2BP1/2/3) were differentially upregulated in HCC. Considering the transcriptional function of FOXM1 as a transcription factor regulating gene transcription, we explored the interaction of FOXM1 with the DNA promoter regions of these three genes in CHIP-seq data of FOXM1 in the Huh-7 cell line. Kaplan-Meier curves identified IGF2BP3 as having a prognostic value in hepatocellular carcinoma. Further analysis revealed five potential FOXM1 binding sequences in its promoter region, which has not been reported.

IGF2BP family proteins are highly expressed during embryonic development and are essential in embryogenesis, while IGF2BP1 and IGF2BP3 are not expressed in normal adult tissues (54). However, in cancer tissues, IGF2BP1 and IGF2BP3 both tested positive. Compared with IGF2BP3, IGF2BP1 has a more complex role in cancer, possessing both pro- and anti-cancer effects, and therefore, IGF2BP3 correlates better with cancer progression (55)., suggesting the importance of further studies on FOXM1 regulation of IGF2BP3. IGF2BP3 can interact with mRNA (53), non-coding RNA (57), and protein (58), respectively. Current studies on FOXM1 and methylation revolve around FOXM1 being methylated, including ALKBH5 demethylation of FOXM1 nascent transcripts (59) and YTHDF1 recognition and binding to m6A-modified FOXM1 mRNA (60), but rarely research has been done on FOXM1 regulating m6a modification. Overall, we predict FOXM1 may affect the messaging of m6a modification by regulating IGF2BP3 transcription.

Metabolic reprogramming is a well-established hallmark of cancer (19). Numerous studies have shown that enhanced glycolysis predicts poor prognosis and promotes tumor progression, immune evasion, and drug resistance in different cancer types (61). The switch from oxidative phosphorylation (OXPHOS) to glycolysis, which is called the “Warburg effect,” is one of the phenomena of cancer (62). Nowadays, targeting the biochemical targets of glycolysis and their potent antagonists or inhibitors with promising anti-cancer effects has become potential therapeutic drug strategies (63). Ketone bodies function as an alternative energy source without glucose, in which fatty acids are mobilized and converted by the liver into ketone bodies to power the body (64). Considering the OXPHOS dysfunction in tumor cells, ketogenic diets (KD) target altered glucose metabolism in cancer cells, further disrupting energy metabolism and adversely affecting tumor cell proliferation (65). In this study, we evaluated the association of FOXM1 with genes related to glycolysis and KBM. FOXM1 expression was positively correlated with most glycolysis-related genes and conversely negatively correlated with KBM-relate genes. By correlation and differential expression analysis, we screened three glycolysis-related genes and five KBM-related genes, indicating that FOXM1 is associated with glycolysis and the KBM process. Among them, we newly analyzed three sequences predicted to bind to FOXM1 in the promoter regions of HK2 and PKM genes, implying that FOXM1 affects the glycolytic biological process in cells by regulating the transcription of HK2 and PKM.

Available studies have shown that there are three critical rate-limiting enzymes in the glycolytic process, namely hexokinase (HK), phosphofructokinase (PFK), and pyruvate kinase (PK), which are essential control nodes of the glycolytic process, and important targets for cancer therapy (66). HK is the first rate-limiting enzyme in glycolysis. The current study found that HK2 is highly expressed in tumors, and its expression level is closely related to the malignancy of the tumor (67). In addition to the glycolytic function of HK2, more and more novel non-classical effects are being discovered. Nuclear-localized HK2 regulates stem/progenitor cell function and differentiation independently of its kinase and metabolic functions (68). Besides, when glycolysis in cellular metabolism is exuberant, HK2 can phosphorylate IκBa in tumor cells, leading to IκBa degradation and NF-κB activation-dependent increase in PD-L1 expression to evade tumor immunity (69). PKM is another rate-limiting enzyme in glycolysis. In mammals, four tissue-specific pyruvate kinases exist, including PKL, PKR, PKM1, and PKM2 (70). The PKM gene forms PKM1 and PKM2 through variable splicing. Unlike constitutively active PKM1, PKM2 is activated only when cellular levels of the allosteric activator increase. Most cancer cells predominantly express PKM2 over PKM1, and PKM2 is mainly found in highly proliferative cells with high anabolic requirements, especially in tumors and embryonic tissues (71). Additionally, the increased expression of PKM2 in tumors was significantly correlated with the prognosis of tumors (72). The involvement of FOXM1 in the glycolytic process has been reported (73, 74). Still, it has not been shown that FOXM1 affects the glycolytic process at the transcriptional level by regulating the expression of HK2 and PKM. This study predicted that FOXM1 binds to the HK2 and PKM promoter regions, providing direction for subsequent studies.

The ceRNA network is a post-transcriptional regulation mediated by miRNA that links the functions of coding and noncoding RNAs. Through the competitive binding of lncRNA or circular RNA to miRNA, the ceRNA network regulates the mRNA expression, potentially affecting the biological process and causing various diseases (75). A previous study has found that FOXM1 plays a core gene in the ceRNA network of HCC (76). In this study, we first searched for miRNAs with experimentally demonstrated FOXM1 interactions and screened them for differential and survival analysis to obtain has-miR-125b-5p. lncRNAs with differential expression and significant prognostic correlation were predicted by pairing has-miR-125b-5p to obtain CYTOR, MIR4435-2HG, and DANCR. Finally, considering that the specific functions of lncRNAs are closely related to their intracellular location and that ceRNA acts mainly in the cytoplasm (17), identified MIR4435-2HG and DANCR as potential target lncRNAs by subcellular localization analysis in the ceRNA network. Has-miR-125b-5p is reported to be downregulated with poor prognosis in HCC patients, and overexpression of has-miR-125b-5p inhibited the proliferation, migration, and invasion of HCC by targeting TXNRD1 (77). MIR4435-2HG was upregulated in HCC, facilitating the progression of liver cancer (78) and promoting cancer cell proliferation (79). Furthermore, DANCR facilitated HCC cell progression by sponging miR-125b-5p through MAPK pathway activation (80). The above studies further suggested the verification of our analysis. In summary, the ceRNA network based on interactions between FOXM1 to hsa-miR-125b-5p to MIR4435-2HG/DANCR was constructed to reveal the gene interaction profile in LIHC.

## Data availability statement

The original contributions presented in the study are included in the article/supplementary material. Further inquiries can be directed to the corresponding authors.

## Ethics statement

The studies involving human participants were reviewed and approved by the Ethics Committee of Changsha Hospital of Traditional Chinese Medicine (Changsha Eighth Hospital). Written informed consent to participate in this study was provided by the participants' legal guardian/next of kin.

## Author contributions

Conceptualization, ZX and HC. Data curation, ZX, CP and KS. Formal analysis, ZX, CP and JY. Funding acquisition, XP and LM. Investigation, YL and YH. Methodology, ZX, CP, HC and BL. Project administration, XP and LM. Resources, ZX, XL and WL. Software, ZX and CP. Supervision, XP and LM. Writing-original draft, ZX and CP. Writing-review & editing, ZX and LM. All authors contributed to the article and approved the submitted version.
